# Metagenomics of the Mucosal Microbiota of European Eels

**DOI:** 10.1128/genomeA.01132-14

**Published:** 2014-11-06

**Authors:** Miguel Carda-Diéguez, Rohit Ghai, Francisco Rodriguez-Valera, Carmen Amaro

**Affiliations:** a Department of Microbiology and Ecology, University of Valencia, Valencia, Spain; b Evolutionary Genomics Group, Departamento de Producción Vegetal y Microbiología, Universidad Miguel Hernández, Alicante, Spain

## Abstract

European eels are an economically important and threatened species that are prone to rapid collapse in farm conditions. Using metagenomics, we show that the eel mucosal microbiota has specific features distinguishing it from the surrounding aquatic community. This is a first step in dissecting the resident microbiota of this critical barrier that may have implications for maintenance of healthy eel populations.

## GENOME ANNOUNCEMENT

The European eel (*Anguilla anguilla*) is a critically endangered catadromous fish whose remarkable migration path involves a transatlantic journey from the European continental waters to the spawning grounds in the Sargasso Sea (1, 2). The larval stage, undergoing metamorphosis, undertakes the journey in reverse, arriving to mainland coastal lagoons and river deltas. Eels breed only in the natural environment, and the entire supply for human consumption comes via eels from young developmental stages captured and reared in fish farms, putting the existing populations at great risk. In fish farms, infection is a leading cause of mortality in these economically important fish (e.g., *Vibrio vulnificus*-induced septicemia) (3). The semipermeable epidermal mucus layer forms the primary protective barrier against the external environment. In this work, we have sequenced the mucosal microbiota metagenome of European eels from five sources: wild adult eels from three locations (Albufera de Valencia, Ebro Delta, and Cabanes-Torreblanca, Spain), farmed eels previously vaccinated against *V. vulnificus* biotype 2, and glass eels (young stage) maintained in a freshwater tank.

Epidermal mucus was obtained, leaving the fish in fishbowls with 1% (vol/vol) saline solution (phosphate-buffered saline [PBS]) for 15 to 20 min, and the resulting mucosal liquid was filtered. The wild eels were returned without damage to their habitats. A series of 5-, 1-, and 0.22-µm polycarbonate filters were used and biomass was retrieved from either the 0.22-µm (Ebro, Albufera, farmed, and glass eels) or the 0.1-µm filter (Ebro and Cabanes-Torreblanca eels), which were treated with 1 mg/ml lysozyme and 0.2 mg/ml proteinase K (final concentrations). DNA extraction was performed as described before (4). Sequencing was performed using Illumina HiSeq (Ebro and Cabanes-Torreblanca eels) (Macrogen, Seoul, South Korea) and 454 (Albufera, farmed, and glass eels) (Centro Superior de Investigación en Salud Pública, Valencia, Spain). The 454 data set sizes are 44 Mb for the farmed eels, 64 Mb for the glass eels, and 41 Mb for the Albufera eels; the Illumina data set sizes are 2.0 Gb for the 0.22-µm filter with the Ebro eels, and 2.2 Gb and 1.0 Gb for the 1.0-µm filter with the Ebro and Cabanes-Torreblanca, respectively. The data sets were analyzed using the MG-RAST server (5).

Regardless of the source of the metagenome, the most dominant organisms were gammaproteobacteria, ranging from at least 30% in the farmed eels to a maximum of nearly 75% in Albufera, making the microbiota very different from that of the surrounding aquatic environment, where *Actinobacteria* are the dominant microbes (6). Several abundant gammaproteobacteria were recognized, e.g., *Pseudomonas*, *Shewanella*, *Stenotrophomonas*, *Vibrio*, and *Aeromonas*, while some betaproteobacteria, like *Comamonas* and *Achromobacter* were also found. Some, like *Pseudomonas*, were found in all samples, while some (*Comamonas*) were found only in farmed and glass eels. *Bacteroidetes* (1.6% to 30%), *Betaproteobacteria* (2.6% to 26%) and *Alphaproteobacteria* (5 to 24%) were other dominant phyla. The similarities of these data sets obtained from dispersed locations strongly suggests the existence of a mucus-specific microbial community that appears quite dissimilar from the planktonic freshwater microbiota. The preservation of such a microbial community may have beneficial effects on eels when they are reared on fish farms.

### Nucleotide sequence accession numbers.

These metagenomes have been deposited in NCBI Short Read Archive (SRA) under the following accession numbers: SRR1578065, SRR1578068, SRR1578098, SRR1580820, SRR1580821, and SRR1580823.
